# Topological Analysis and Recovery of Entanglements in Polymer Melts

**DOI:** 10.1021/acs.macromol.3c00278

**Published:** 2023-04-18

**Authors:** Mattia Alberto Ubertini, Angelo Rosa

**Affiliations:** Scuola Internazionale Superiore di Studi Avanzati (SISSA), Via Bonomea 265, 34136 Trieste, Italy

## Abstract

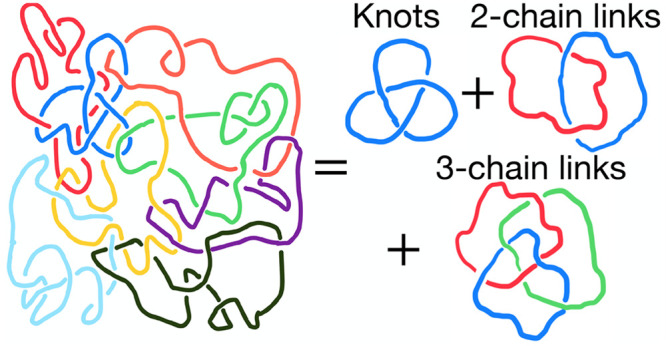

The viscous flow of polymer chains in dense melts is dominated by topological constraints whenever the single-chain contour length, *N*, becomes larger than the characteristic scale *N*_e_, defining comprehensively the macroscopic rheological properties of the highly entangled polymer systems. Even though they are naturally connected to the presence of hard constraints like knots and links within the polymer chains, the difficulty of integrating the rigorous language of mathematical topology with the physics of polymer melts has limited somehow a genuine topological approach to the problem of classifying these constraints and to how they are related to the rheological entanglements. In this work, we tackle this problem by studying the occurrence of knots and links in lattice melts of randomly knotted and randomly concatenated ring polymers with various bending stiffness values. Specifically, by introducing an algorithm that shrinks the chains to their minimal shapes that do not violate topological constraints and by analyzing those in terms of suitable topological invariants, we provide a detailed characterization of the topological properties at the intrachain level (knots) and of links between pairs and triplets of distinct chains. Then, by employing the Z1 algorithm on the minimal conformations to extract the entanglement length *N*_e_, we show that the ratio *N*/*N*_e_, the number of entanglements per chain, can be remarkably well reconstructed in terms of only two-chain links.

## 1 Introduction

The viscoelastic behavior of concentrated solutions or melts of *linear* polymer chains can be understood assuming^1−3^ slow reptative flow of each chain through the network of topological obstacles (entanglements) formed by the surrounding chains. According to this picture, entanglements confine each chain within an effective tube-like region of diameter , where ⟨*b*⟩ is the mean bond length, *n*_K_ is the Kuhn length of the polymers (in monomer units^4^) accounting for the fiber stiffness, while the *topological* entanglement length *N*_e_ is the characteristic, material-dependent,^5−7^ length scale marking the crossover from *non-entangled* to *entangled* polymer behavior. Then, the mean size or radius of gyration ⟨*R*_g_⟩ of polymer chains with contour length *N* ≳ *N*_e_ follows the power-law behavior
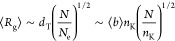
1and all of the essential structural and dynamical information about the melt can be understood in terms of the single parameter *N*_e_. Although, in general, estimating *N*_e_ is a challenging problem,^5,8^ considerable progress has been made (at least in numerical simulations) in terms of *primitive path analysis*(9−12) (PPA). By exploiting the simple yet ingenious idea^2^ that linear chains can be “coarse-grained” down to their minimal path without violating the topological constraints, PPA provides an intuitive understanding of the microscopic^13^ nature of entanglements.

Alternatively, polymeric entanglements may be also modeled as *physical links* between chains.^12,14−22^ Specifically, the idea is “to map” the system of entangled chains to an equivalent one of randomly entangled (namely, self-knotted and linked) ring polymers and employ suitable *topological invariants*(23) to identify and then classify, in a mathematically rigorous manner!, the total amount of entanglements of the melt and connect them to the macroscopic viscoelastic behavior.

The connection between the two pictures is, however, not that straightforward. The main reason is that the complete statistical–mechanical classification of a polymer melt would require an *infinite set*(16,18) of topological invariants in terms of pairs, triplets, etc., of loops, not to mention that analytical theories are mathematically hard^24^ and their applicability to dense systems is limited.

Motivated by these considerations, we rethink the problem of characterizing a melt of entangled polymer chains in terms of topological invariants and outline, in a quantitative manner, the connection between the latter and the topological entanglement length of the chains. More specifically, we perform extensive computer simulations of *randomly knotted* and *randomly concatenated* ring polymers under dense conditions and different values of the bending stiffness of the polymer fiber as models for entangled polymer systems.

**Figure 1:**
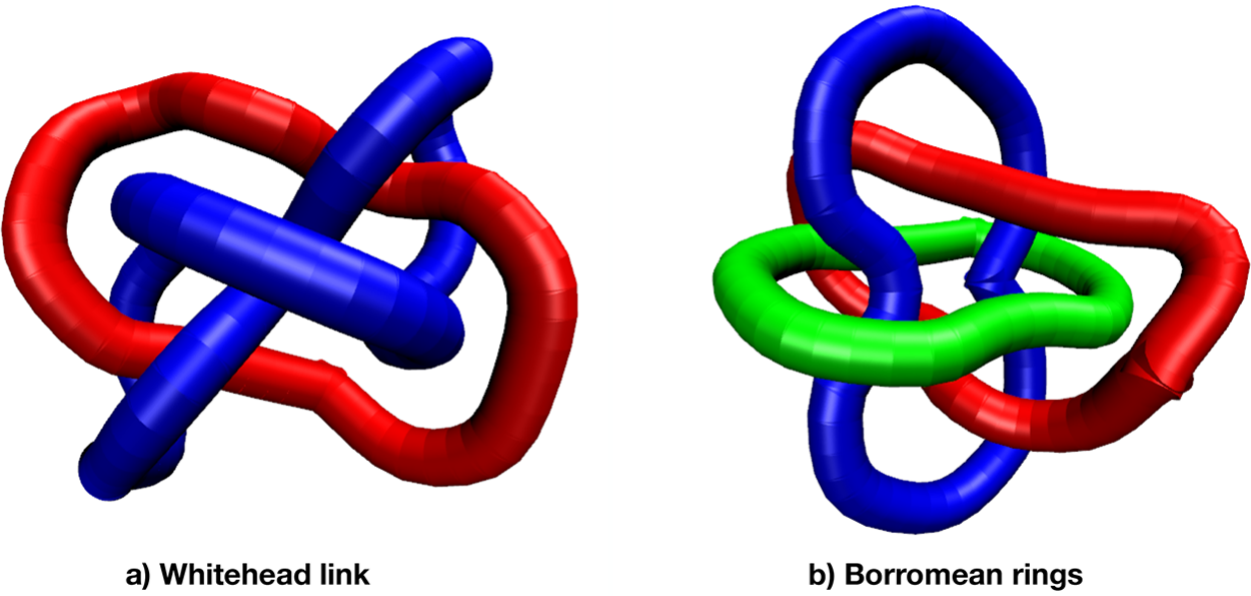
Examples of ring polymer structures with Gauss linking number [GLN (see eq 3)] equal to 0. (a) Two rings intertwined in the Whitehead link 5_1_^2^. (b) Three rings clustered into the Borromean conformation 6_2_^3^. Both conformations have been extracted from numerical simulations of ring polymer melts after the minimization procedure described in the text. To name the conformations here and in the rest of the text, we have used the classical nomenclature introduced in Rolfsen’s book (see section 2.3).

Then, inspired by PPA and the recent work of Bobbili and Milner^21^ on molecular dynamics simulations of melts of randomly linked ring polymers, we construct an algorithm for *contracting* the contour length of each ring in the melt to its “primitive” or “minimal” length that does not violate the topological constraints with the other rings. The conformational properties of the primitive ring structures are thus explored at the single-ring level (knots), between any rings’ pair (see the Whitehead link in Figure 1a), and between any rings’ triplet (see the complex Borromean configuration in Figure 1b). By looking at the relative abundance of these topological structures as a function of the bending stiffness of the polymers, we combine them into a proxy for the quantitative prediction of the number of entanglement lengths, *N*/*N*_e_, of the polymers.

The paper is structured as follows. In section 2, we present some technical details of the lattice polymer model, explain the shrinking algorithm developed for the calculation of the ring minimal path, introduce the notation and the topological invariants for the characterization of knots and links, and, finally, illustrate the idea behind the Z1 algorithm used for the calculation of the entanglement length. In section 3, we present the main results of our work, while in section 4, we provide some discussion and conclusions regarding the connection between knots, two-chain links, three-chain links, and the entanglement length of the polymers. Additional figures are included in the Supporting Information.

## 2 Model and Methods

### 2.1 Polymer Model

Model systems of *M* randomly knotted and concatenated ring polymers of *N* monomers each were prepared on the basis of the kinetic Monte Carlo (kMC) algorithm illustrated in refs (25) and (26) and closely related to other models that have appeared in the literature.^27−30^ The polymer model, which is defined on the three-dimensional face-centered-cubic (fcc) lattice of unit step = *a*, accounts for (i) chain connectivity, (ii) bending stiffness, (iii) excluded volume, and (iv) topological rearrangement of the polymer chains. The kinetic algorithm consists of a combination of Rouse-like and reptation-like moves for chain dynamics that take advantage of a certain amount of stored contour length along the polymer filament that simplifies the process of chain equilibration. As a consequence, the polymers are locally elastic, with fluctuating monomer–monomer bonds of mean length ⟨*b*⟩ implying that the effective polymer contour length is *N*⟨*b*⟩.

Ring conformations were equilibrated through long runs at the average monomer number per lattice site of  or unit volume of  corresponding to melt conditions. By modulating the Kuhn segment *n*_K_ through the bending penalty Hamiltonian , where κ_bend_ is the bending stiffness and θ_*i*_ is the angle between two consecutive bonds along the chain, one can show^26^ that chains become locally stiffer. Table 1 summarizes (i) mean bond length ⟨*b*⟩, (ii) mean cosine value ⟨cosθ⟩ between two consecutive bonds along the chain, (iii) and Kuhn length *n*_K_, as a function of κ_bend_. The simulation box of linear size *L*_box_ has periodic boundaries for the enforcement of bulky melt conditions. By fixing the total number of monomers to the convenient value of 134 400, we have *L*_box_/*a* = 30√2. In this paper, we have studied polymer melts with *N* × *M* = (40 × 3360, 80 × 1680, 160 × 840, 320 × 420, 640 × 210).

**Table 1:** Values of Physical Parameters for the Ring Polymer Melts Investigated in This Paper^a^

κ_bend_/(*k*_B_*T*)	⟨*b*⟩/*a*	⟨cosθ⟩	*n*_K_
0	0.733	0.186	1.965
1	0.695	0.455	3.157
2	0.663	0.638	5.118

a *a* is the unit distance of the fcc lattice, and the monomer number per unit volume is equal to  (see the text and ref (26) for details): κ_bend_, bending stiffness in statistical–mechanical thermal units *k*_B_*T*, where *k*_B_ is the Boltzmann constant and *T* is the temperature; ⟨*b*⟩, mean bond length;^31^ ⟨cosθ⟩, mean cosine value between two consecutive bonds along the chain;^31^*n*_K_, Kuhn length.^32^.

As illustrated in ref (25), we introduce random strand crossing between nearby polymer strands at the fixed rate of one per 10^4^ kMC elementary steps. In this way, we induce the violation of the topological constraints and obtain equilibrated melts of rings with intrachain (i.e., knots) and interchain (i.e., links) nontrivial and randomly generated topologies. By construction then, the algorithm generates rings with *annealed* topologies; in other words, our ring conformations represent a thermodynamic ensemble of melts of randomly knotted and concatenated rings at the given density for different polymer lengths *N* and bending rigidities κ_bend_. To ensure proper system equilibration as well as accurate polymer statistics,^33^ the total computational cost of the simulations goes from 2 × 10^6^ τ_MC_ for *N* = 40 and κ_bend_/(*k*_B_*T*) = 0 to 7 × 10^7^ τ_MC_ for *N* = 640 and κ_bend_/(*k*_B_*T*) = 2. Here, τ_MC_, the MC “time” unit,^25,26^ is equal to *N* × *M* kMC elementary steps.

Violation of topological constraints by random strand crossing induces a massive reorganization of the statistics of polymer chains. As studied in ref (25), while unknotted and nonconcatenated rings remain compact with asymptotic mean gyration radis following the power law
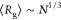
randomly knotted and randomly linked melt of rings swell as
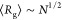
i.e., locally they become equivalent to melts of linear chains (see eq 1 and Figure S1). Furthermore, the distinctive anticorrelation of the bond-vector correlation function
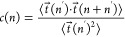
2as a function of the effective monomer length separation, *n*, along the chain reported^26,34^ in melts of unknotted and nonconcatenated rings disappears in randomly linked systems (see Figure S2), whose behavior is close to that for linear chains (see the dashed lines). Overall, we may conclude that randomly linked rings reproduce the essential features of entangled linear polymer chains in a melt. Next, we will use these systems to investigate the microscopic nature of entanglements by means of the rigorous language of topological invariants.

### 2.2 Algorithmic Pipeline to Rings Minimal Paths

To detect and classify topological interactions in equilibrated melts of entangled rings, we introduce a simple “shrinking” algorithm that takes explicit advantage of the presence of stored lengths along the contour length of each chain. Specifically, the algorithm consists of iterating the following steps: (1) We remove all of the stored lengths from the polymers. Of course, this excision process leads to a reduction in the total contour length of each chain. Notice that, by construction, this does not lead to violations of the topological constraints, neither intrachain ones (such as knots, for instance) nor between different chains (i.e., links). (2) After the excision, we perform a short MC run (on the order of 10–100 τ_MC_) under global preservation of topological constraints (i.e., without strand crossing). In general, this step leads to the formation of new units of stored length that, in turns, will be removed by the next implementation of step 1, and so on. We then apply these operations, individually, to single chains (section 3.1), pairs of chains (section 3.2), and triplets of chains (section 3.3). In all of these cases, the procedure stops when the number of monomers of each shrinking chain has not changed for 300 consecutive iterations; in this case, we assume that each chain has reached its *minimal* shape.

To validate the algorithm, we have tested it first on the “trivial” case of unknotted and nonconcatenated ring polymers in a melt. We have thus verified that shape minimization of rings taken one by one or simultaneous application of the procedure on the whole melt led to what is expected on the basis of intuition, that individual rings shrink to single points. Then, by our algorithm, we may isolate unknotted and nonconcatenated configurations from those with nontrivial topologies.

### 2.3 Classification of Knots and Links

Following the contour length simplification outlined in section 2.2, we have investigated the statistical abundance of the following topological objects: (i) knots in single-ring polymers (section 3.1), (ii) links between pairs of ring polymers (two-chain topological structures) (section 3.2), and (iii) links between triplets of ring polymers (three-chain topological structures) (section 3.3). We do not proceed beyond step (iii) because, although in principle the procedure can be applied to even larger groups of rings, the factorial growth of possible combinations makes the analysis tediously lengthy from a computational point of view. On the contrary, we will show (section 3.4) that this is perfectly adequate to capture the entanglement length *N*_e_.

#### 2.3.1 Notation

In referring to a given knot or link, we follow the standard convention as explained in the book by Rolfsen.^35^ Namely, a knot or a link is defined by the symbol *K*_*i*_^*p*^, where *K* represents the number of irreducible crossings of the knot (or the link), *p* is the number of rings that take part in the topological structure (e.g., *p* = 2 for links between two rings) and *i* is an enumerative index assigned to distinguish topologically inequivalent structures with the same *K* and *p*. For knots in single rings, *p* = 1 is tacitly assumed and, as an example, the simple trefoil knot is identified by Rolfsen’s symbol 3_1_.

#### 2.3.2 Topological Invariants

Nontrivial knots and links can be detected and hence classified by means of suitable *topological invariants*.^23,36^ In this work, we resort to the method of the so-called *Jones polynomials*(37) that assign to each knot a distinctive algebraic polynomial. Specifically (section 3.1), we use the implementation of the Jones polynomials featured in the Python package *Topoly*(38) to recognize and categorize knots within single-ring polymers and, in this way, benchmark the simplification algorithm of section 2.2.

Moreover, and as for links alone,^39^ we also consider the simpler Gauss linking number (GLN):
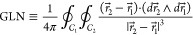
3which gives the number of times two closed loops  and , parametrized by coordinates *r⃗*_1_ and *r⃗*_2_, respectively, wind around each other. While intuitive and easier to compute with respect to the Jones polynomials, GLN has nonetheless severe limitations.^36^ It is in fact widely known that, while GLN ≠ 0 means that the two rings are linked, the opposite (GLN = 0) is not necessarily true. Take for instance the example shown in Figure 1a, i.e., the so-called Whitehead link 5_1_^2^, constituted by two irreducibly linked rings and yet GLN = 0. In addition, one may imagine even more complex situations such as the one displayed in Figure 1b (the so-called Borromean conformation 6_2_^3^) in which three rings, which are two-by-two nonconcatenated, are irreducibly linked. Such structures are, obviously, also not detected by eq 3. In the course of the paper (section 3), we will show how these structures (which elude eq 3) can be properly detected and, then, how to quantify their impact on the entanglement properties of the melt.

### 2.4 Calculation of the Entanglement Length

By following the approach by Bobbili and Milner^21^ for molecular dynamics simulations of a melt of seemingly shrunk and randomly linked ring polymers, we estimate *N*_e_ by applying a recent version (Z1+^40^) of the Z1 algorithm.^11,41−43^ The Z1 algorithm consists of the implementation of a series of geometrical operations that transform the entangled polymer chains in a collection of straight segments that are sharply bent at the entanglement points, and then one may estimate *N*_e_ as the average length of these straight segments. In particular, the Z1+ version takes explicitly into account the role of chain self-entanglements (knots) during the determination of *N*_e_. The effects of it will be discussed in section 3.4.

## 3 Results

In this section, we will describe results concerning the appearances of knots (section 3.1) and links (sections 3.2 and 3.3) in melts of entangled randomly linked rings of different chain length and bending stiffness values. Then (section 3.4), we will show how to establish a direct connection between the topology of links and the entanglement length of the chains. While we have considered different chain lengths (section 2.1), covering the full crossover from loosely to strongly interpenetrating polymers, for the sake of brevity we will present many results for only the most representative and longest chains with *N* = 640.

### 3.1 One-Chain Topological Structures, Knots

**Figure 2:**
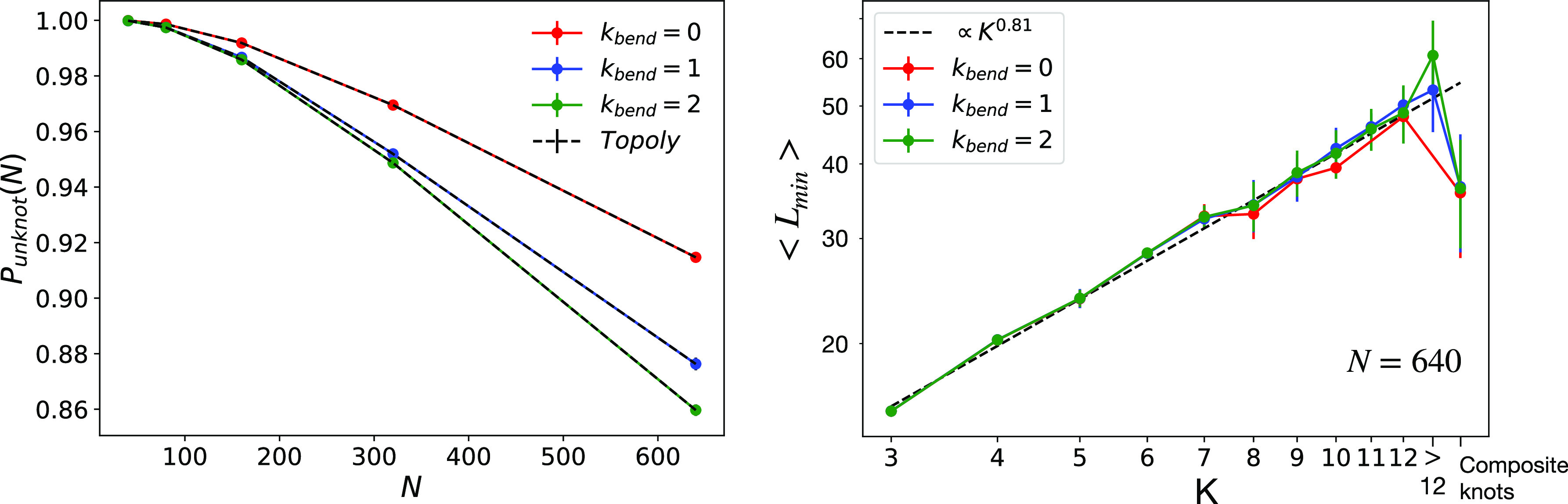
*P*_unknot_ (left), probability that a ring is unknotted as a function of the number of monomers, *N*, and for different bending stiffness values, κ_bend_. The shrinking algorithm (solid lines) and *Topoly* (dashed lines) are in perfect agreement. ⟨*L*_min_⟩ (right), average minimal contour length of rings with *N* = 640 monomers as a function of knot crossing number, *K*, and for different bending stiffness values, κ_bend_. Each error bar corresponds to the standard deviation calculated for the ring population at the respective crossing number *K*. The data are described well by the simple power-law behavior ∼*K*^0.81^ (dashed line). The generic label “>12” follows from the fact that *Topoly* is unable^38^ to recognize properly knots with >12 crossings.

First, we have applied our algorithm (section 2.2) to detect knots in single rings, and to prove its reliability, we have applied the *Topoly* tool (section 2.3.2) to the simplified ring shape to classify the relative knot type. As a result, we have always found a nontrivial Jones polynomial corresponding to those rings that do not shrink to a point; in other words, the shrinking algorithm recovers knots successfully and the results map one to one to those obtained by *Topoly* [see Figure 2 (left panel) for the probability *P*_unknot_ that a ring is unknotted as a function of monomer number *N* and at different bending stiffness κ_bend_]. Overall, *P*_unknot_ is always a decreasing function of polymer length *N*, a result in line^44,45^ with other generic polymer models. At the same time, for a fixed *N*, *P*_unknot_ decreases as a function of κ_bend_ or stiffer rings are more likely to form knots with respect to more bendable ones, and this difference appears to increase with *N*. This feature also seems to be quite general having been reported recently^46^ in the context of computer simulations of isolated semiflexible ring polymers. Notice, however, that the probability of observing a knot remains small [for κ_bend_/(*k*_B_*T*) = 2 and *N* = 640, this is only 1 – *P*_unknot_ ≈ 14%]. Again, this is in qualitative accord with ref (46), although knots here seem slightly more likely (1 – *P*_unknot_ ≲ 5% in ref (46)): arguably, this is a consequence of considering polymers under melt conditions and not isolated chains.

While Jones polynomials (as well as any other topological invariant) inform us about the knot type “trapped” within the ring, by our shrinking algorithm we may also quantify the “amount” of topological entanglement “stored” within a nontrivial knot in terms of the corresponding “minimal” contour length. In particular, rings hosting “simpler” knots (i.e., low-crossing knots) shrink more and occupy less primitive length in comparison to more complicated knots. To show this, we have computed the mean value, ⟨*L*_min_⟩, of the ring minimal contour length as a function of crossing number *K* characterizing the hosted knot. In principle, the ring minimal contour length is a random quantity because the shrinking procedures are performed stochastically; on the contrary, we see that these fluctuations are, for each knot type, comparably small (Figure S3); i.e., the minimization procedure converges to a well-defined minimal shape. Notably, ⟨*L*_min_⟩ is a genuine topological signature; it is almost insensitive to bending stiffness κ_bend_ [see Figure 2 (right panel)] and increases with characteristic power-law *K*^α^ with α ≃ 0.81 (dashed line). Interestingly, the same power-law behavior in relation to the scaling of the minimal rope length required to tie a nontrivial knot into a flexible rope has been reported recently.^47^ We conclude that, for a given knotted ring, our minimization algorithm converges to the corresponding minimal knot structure. Moreover, and again in agreement with ref (47), we find that the so-called *alternating* knots, knots where crossings alternate under and over when moving along the filament, display larger ⟨*L*_min_⟩ values and are less frequently seen (Figures S3 and S4, respectively, for *K* ≥ 8 only^48^) than the *non-alternating* ones for the same number of crossings.

### 3.2 Two-Chain Topological Structures, Links

After having investigated the amount of knots, we turn our attention to the topological interactions between pairs of rings. For this purpose, we have devised the following way to distinguish between those links that have GLNs (eq 3) not equal to zero and links with GLNs equal to zero [such as the Whitehead link (see Figure 1a)]. A link between two closed chains with a GLN of zero can be unlinked by performing a certain number of crossings between strands of the same chain, while those with GLNs not equal to zero cannot be simplified and would remain linked. According to that, we have applied the shrinking procedure to the two rings in two distinct manners: (i) straightforwardly as described in section 2.2 and (ii) with intrachain crossing allowed. In this way, the excess of links between pairs of rings with GLNs of zero can be measured as the “difference” between manners i and ii. To test the robustness of this procedure, we have computed the corresponding Jones polynomial for the linked rings that display GLNs of zero. In the end, it turns out that only the pairs of rings that emerge as non-trivially linked feature nontrivial Jones polynomials as well.

**Figure 3:**
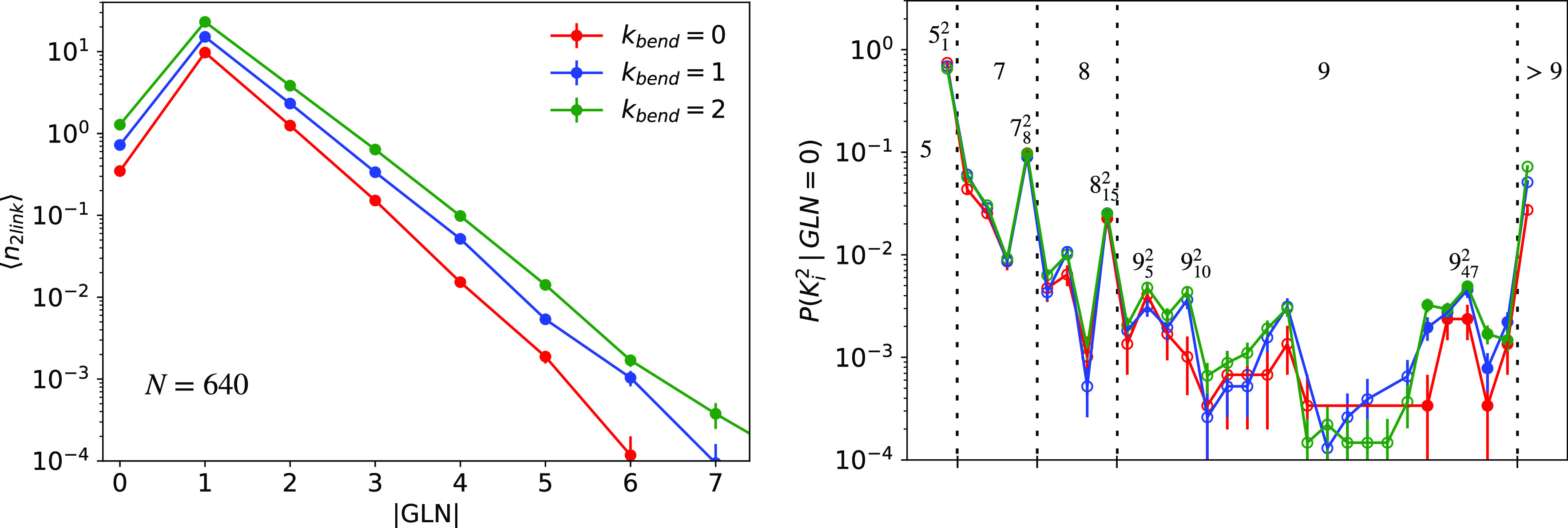
⟨*n*_2link_(|GLN|)⟩ (left), mean number of two-chain links per ring as a function of absolute Gauss linking number |GLN|. *P*(*K*_*i*_^2^|GLN = 0) (right), fractional population of two-chain links *K*_*i*_^2^ (termed according to Rolfsen’s convention^35^) having a GLN of zero. Here, as well as in the right panel of Figure 4 and Figure S4, error bars are estimated by assuming the formula for simple binomial statistics for the probability of observing a given link (knot, in Figure S4) type in the total population. Empty and filled circles represent data for alternating and non-alternating links, respectively, while vertical dotted lines separate link classes with the same number of crossings. The displayed link labels correspond to those links appearing with the highest frequency in their class of number of crossings *K*. The generic label “>9” follows from the fact that *Topoly* cannot^38^ recognize properly links with >9 crossings. In both panels, data refer to rings with *N* = 640 and different bending stiffness values, κ_bend_.

The mean number of links per chain with the absolute Gauss linking number, *n*_2link_(|GLN|), for rings with *N* = 640 and different bending stiffness values is shown in the left panel of Figure 3 and Figure S5 for the other polymer lengths. We find that links are mainly simple Hopf links (i.e., |GLN| = 1), while links with a GLN of zero are rare and have a frequency between those for |GLN| = 2 and |GLN| = 3. More complex links follow an exponentially decaying distribution, in agreement with ref (25). Finally, many possible types of non-equivalent links exist for GLNs of zero, and we have further investigated, by the Jones polynomials, which structures emerge and their relative abundance (Figure 3, right panel). As one may see, polymer conformations are dominated by the Whitehead link (Rolfsen’s symbol 5_1_^2^) that, of course, is the simplest one in terms of crossings. Nonetheless, we report a remarkably complex spectrum of link types that is affected very little by the bending stiffness of the chains. In particular, with at least seven crossings, we find that the most abundant links turn out to be the non-alternating ones with probabilities significantly higher than those of the alternating ones. The only notable exception is for nine crossings, where the non-alternating 9_47_^2^ occurs with the same frequency as 9_5_^2^ and 9_10_^2^, which are indeed alternating; overall, though, all of these links are very rare.

### 3.3 Three-Chain Topological Structures, Links

We consider now topological structures between ring triplets. To fix the ideas, we notice that three-chain links can be grouped as follows. One group consists of those links that can be “reduced” in terms of the “composition” of simpler two-chain structures like those seen in section 3.2, while the second group consists of the others that can be then called *irreducible*. Those belonging to the first group are (a) *poly(3)catenanes*, chains made of three rings in which two nonconcatenated rings are connected to a common ring, and (b) *triangles*, triplets of rings that are concatenated in a two-by-two manner. Because of the detection of pairwise links (section 3.2), their presence can be efficiently assessed. The presence of these structures has been amply documented in melts of concatenated rings;^49^ in particular, they can be identified, subject to the limitations discussed in section 3.2, via the summation of pairwise concatenations and the relative GLN. On the contrary, irreducible three-chain links, which cannot be detected by decomposition into pairwise links, can be divided further into two classes: (c) *poly(2)catenane+1-ring*, structures made of a poly(2)catenane (i.e., a pair of concatenated rings) and another ring that is not directly concatenated (in a pairwise manner) with any of the twos, and (d) *Brunnian* links, nontrivial links that become a set of trivial links whenever one component ring is unlinked from the others (the Borromean conformation in Figure 1b constitutes the simplest example).

**Figure 4:**
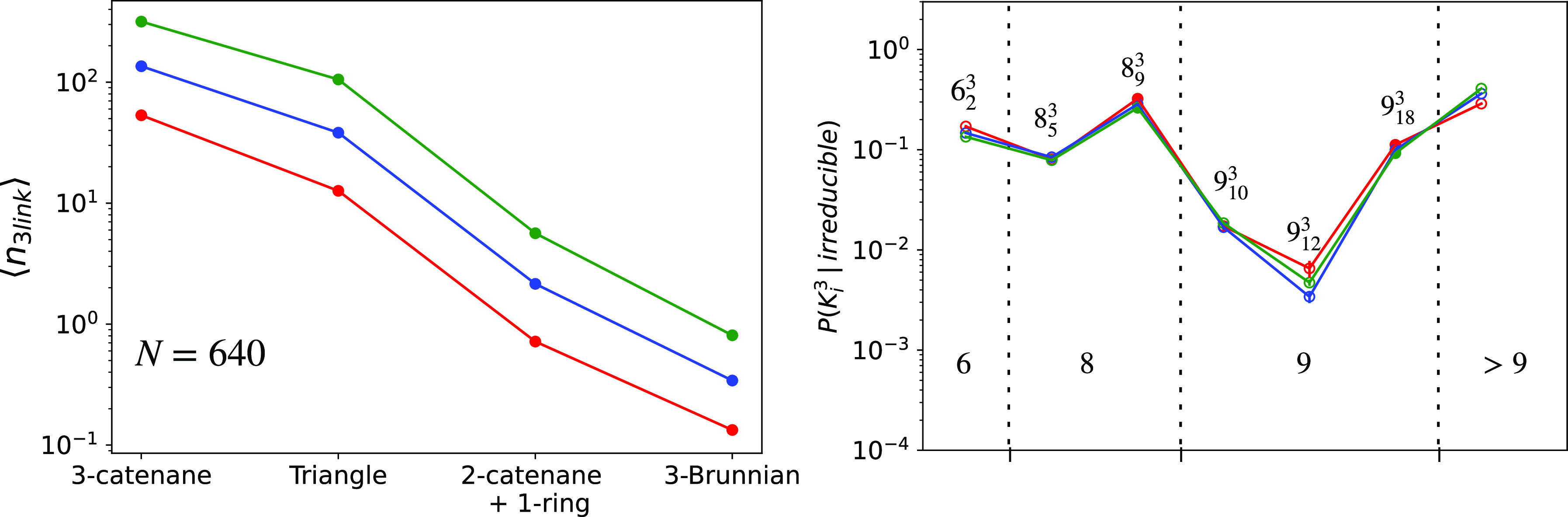
⟨*n*_3link_⟩ (left), mean number of different three-chain structures per ring. *P*(*K*_*i*_^3^|irreducible) (right), fractional population of three-chain links *K*_*i*_^3^ (termed according to Rolfsen’s convention^35^) belonging to the poly(2)catenane+1-ring and Brunnian classes (see the text for details). These are “irreducible” with respect to the simpler compositions of two-chain links. As in Figure 3, empty and filled circles represent data for alternating and non-alternating links, respectively, while vertical dotted lines delimit link classes with the same number of crossings. Similarly, the generic label “>9” follows from the fact that *Topoly* cannot^38^ recognize properly links with >9 crossings. In both panels, data refer to rings with *N* = 640 and different bending stiffness values, κ_bend_.

To characterize the relative abundance of each of these structures, we have studied the mean number of different three-chain links per ring, ⟨*n*_3link_⟩. We find (Figure 4, left panel) that links are present maximally in poly(3)catenane and triangle structures, yet, although rarer, the other two classes appear in detectable amounts. Notably, as for single knots and two-chain links (left panels of Figures 2 and 3), the abundance of three-chain structures increases with chain stiffness. As for the links, within classes (c) and (d), we have analyzed the different topological inequivalent concatenated structures with *Topoly*. Due to the complexity of the analyzed structures, *Topoly* cannot classify them properly in ∼50% of the cases after nine crossings. As for the successfully determined links (Figure 4, right panel), we find that the most abundant links are 6_2_^3^ (i.e., Borromean rings) and 8_9_^3^ (which belongs to class (c)). Again, at a fixed number of crossings, the most abundant structures are the non-alternating ones (8_5_^3^, 9_10_^3^, and 9_12_^3^ are all alternating), thus highlighting the preference for non-alternating linked structures.

### 3.4 Quantitative Connection to Entanglement Length *N*_e_

**Figure 5:**
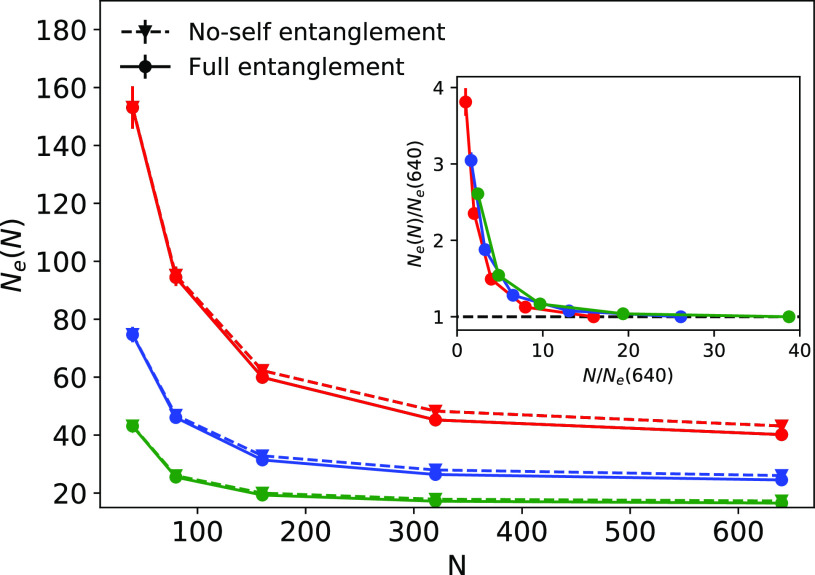
Entanglement length, *N*_e_, as a function of the number of monomers per chain, *N*, for different bending stiffness values, κ_bend_. Solid and dashed lines depict data after including and removing, respectively, self-entanglements (knots) through the Z1 algorithm (technical details in section 2.4). The inset shows the *x* and *y* coordinates of data with self-entanglements normalized by the corresponding asymptotic value, *N*_e_(*N* = 640), of the entanglement length.

By applying the shrinking algorithm to the whole melt, we take topological interactions of any order into account, and finally, we can assess their contribution to topological entanglement length *N*_e_ (eq 1). In general, the process of shrinking reduces the contour length of each ring inasmuch as the topological constraints allow. Thus, if a ring is unknotted and nonconcatenated, it will shrink to a point and will be not taken into account because it is assumed not to be contributing to the entanglement length of the chains.^50^ Conversely, the more the rings are entangled, the less they will shrink. Then we apply (see section 2.4 for details) the Z1 algorithm^11,40−43^ to the shrunken structures and estimate *N*_e_ thereby. Figure 5 (main panel, solid lines) shows the values of *N*_e_ as a function of *N* and for different bending stiffness values, κ_bend_. In all cases, *N*_e_(*N*) tends to an asymptotic value {*N*_e_ = [40.(2), 24.(5), 16.(5)] for *N* = 640 and for κ_bend_/(*k*_B_*T*) = 0, 1, and 2, respectively}. Interestingly, by rescaling both *x* and *y* coordinates by the corresponding asymptotic value, we find the distinct curves collapse onto each other (Figure 5, inset).^51^ Notice also that the characteristic large values of *N*_e_ measured at small values of *N* are due to the fact that rings are loosely linked; in contrast, at larger values of *N* rings turn out to be concatenated into a single percolating network of concatenated rings (see Figure S6).

While, not surprisingly,^26^*N*_e_ decreases as polymers become stiffer, it is worth comparing these values to those {*N*_e_ = [80.37(9), 29.76(4), 13.08(8)]} obtained by us^26^ by applying theoretical results based on PPA. When κ_bend_/(*k*_B_*T*) = 0, the Z1 value is twice the PPA one. This discrepancy was noticed previously^13,43,52^ and explained as a consequence of orientational correlations between subsequent primitive path segments. Interestingly the discrepancy almost disappears in semiflexible melts for which κ_bend_/(*k*_B_*T*) = 1 and 2, suggesting that the corresponding correlations are limited to polymer chains that are quite flexible on the entanglement scale (loosely entangled^53^). With respect to the possible role of self-entanglements (i.e., knots), they influence *N*_e_ only marginally (compare solid and dashed lines in Figure 5), in agreement with the result (section 3.1) that only a small fraction of the rings (≈10%) display knots. Nonetheless, when compared to the similar analysis published in ref (10) on the role of knots in entangled melts of linear polymers, the differences reported by us here appear [especially for the more flexible case κ_bend_/(*k*_B_*T*) = 0] to be slightly stronger. A likely explanation for this result is, as already^51^ pointed out, the possible role of the ring closure. In fact, we will see (discussion in section 4) that linear chains of comparable length are significantly less knotted than their ring counterparts.

**Figure 6:**
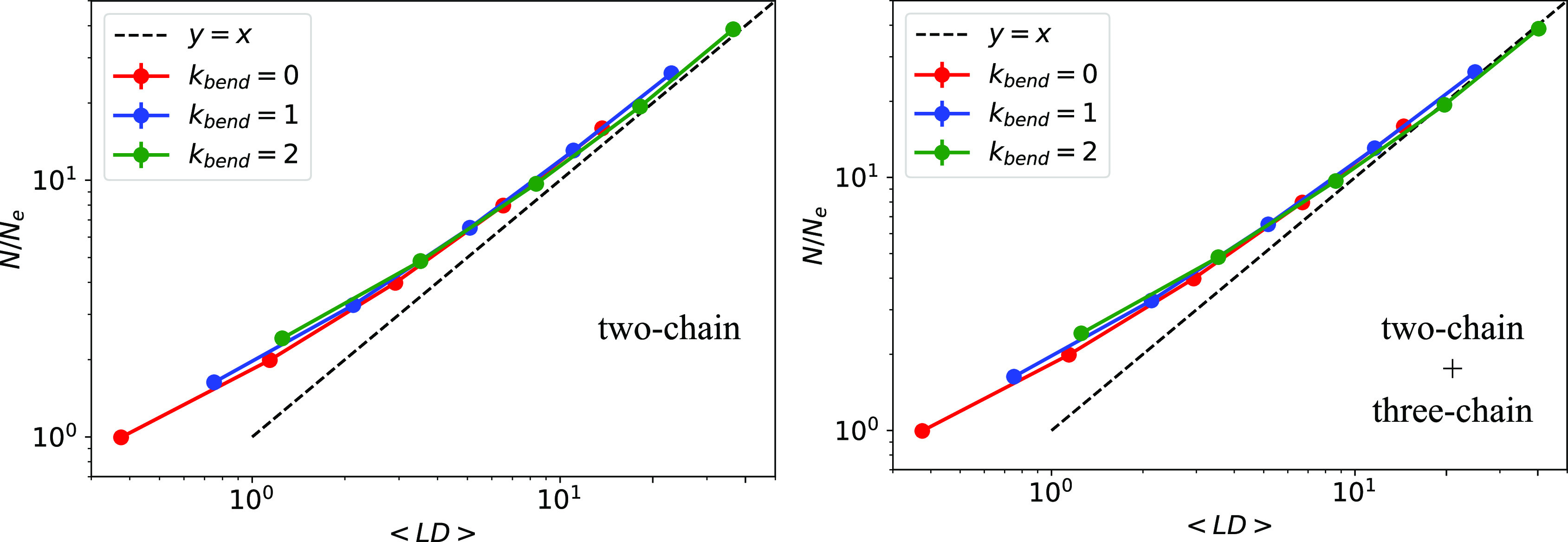
Number of entanglements per ring, *N*/*N*_e_, as a function of the mean linking degree, ⟨LD⟩, computed (see eq 4) by taking into account the contribution from two-chain links solely (left) and after including (right) also the contribution of three-chain links.

Finally, we show how to connect, in a quantitative manner, *N*_e_ to the linking properties of the rings (see sections 3.2 and 3.3). For this purpose, we define the ring mean linking degree ⟨LD⟩ as
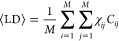
4where each sum runs over the total number of chains [*M* (see section 2.1)] in the melt. *C*_*ij*_ is the *M* × *M* matrix expressing the concatenation status between rings *i* and *j* and is defined as
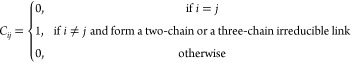
5The “weight” factor χ_*ij*_ takes into account the “complexity” of the two-chain (section 3.2) and three-chain (section 3.3) links: (i) for two-chain links, χ_*ij*_ = |GLN| or  depending on whether GLN ≠ 0 or GLN = 0, respectively; (ii) for three-chain links, . Here, *K* is the number of crossings characterizing the link; in other words, each crossing of the link contributes ^1^/_2_ to an entanglement point. Figure 6 (left panel) shows that, by taking into account only the contribution of two-chain links and in the large-chain limit, eq 4 accounts remarkably well for the number of entanglements, *N*/*N*_e_, of each chain. Further inclusion (right panel) of three-chain links adds only a small contribution; otherwise, it does not improve the agreement significantly. This is probably the most important result of this work. It says that two-chain links alone capture almost completely the nature of entanglement length *N*_e_ and that, through eq 4, a true quantitative connection between them can be established.

## 4 Discussion and Conclusions

Understanding the microscopic nature of topological constraints in melts of polymer chains is a long-standing, classical^5,8,15,16^ problem in soft matter physics. In this work, we have characterized accurately the topological state of melts of randomly knotted and concatenated ring polymers used as models for (long) linear polymer systems and, then, shown its relationship with entanglement length *N*_e_ of the chains, which is the central quantity of any rheological theory.^1−3^

To accomplish the task, we have first shrunk the chains to their “minimal shape” by introducing a simple numerical algorithm that chops off progressively the contour length of the chains without producing any violation of the topological constraints present in the systems. Then, we have systematically carried out an analysis of the rings’ topology from the single-chain (knots) to two- and three-chain (links) levels.

By using the Jones polynomials as suitable topological invariants, we have characterized the topological spectrum as a function of the bending stiffness of the chains by finding, in particular, that stiffer rings are more knotted and more concatenated than more flexible ones (Figures 2–4). We have also found that, quite systematically, for both knots and links non-alternating structures are more likely to be present with respect to the alternating ones (at the same topological complexity). By applying the Z1 algorithm to the shrunken structures, we have computed the entanglement length *N*_e_ of the melts for the different stiffnesses values and found that chain self-entanglements (knots) do not play a significant role in *N*_e_ (Figure 5), in fair agreement with the fact that rings are rarely knotted (Figure 2). Most importantly, we have demonstrated (Figure 6) that the ring mean linking degree ⟨LD⟩, which accounts for the mean number of entanglement points of each chain in the melt, is a prior for the number of entanglements *N*/*N*_e_ that points to a nontrivial connection between the topology of the chains and the rheological entanglement of the system. Interestingly, the quantitative matching between ⟨LD⟩ and *N*/*N*_e_ is already remarkably accurate upon inclusion of only the contributions up to the simplest two-chain linked structures, suggesting that, at least for the chain lengths examined here, links of higher order contribute negligibly. Overall, these findings highlight the connection between the rheological entanglements and the topological links between distinct chains acting at the microscopic level.

We conclude by discussing more carefully our assumption (see section 1) that ring melts can be used to understand entanglement in linear melts. For this purpose, we have analyzed the occurrence of knots and links in melts of linear chains with *N* = 320^54^ and for the same physical parameters (i.e., density and bending stiffness) employed for ring melts. The results for the unknot probability [*P*_unknot_ (see also the left panel of Figure 2)] and the mean number of two-chain links with absolute Gauss linking number |GLN| [⟨*n*_2link_(|GLN|)⟩ (see also the left panel of Figure 3)], in comparison with the analogous ones for rings, are reported in Figure S7 (top and bottom rows, respectively). For the same *N* = 320, knots are clearly less abundant in linear than in ring melts, and we ascribe this to the closure constraint that may enhance the formation of knots in rings compared to linear chains. On the contrary, two-chain links for which |GLN| = 1 [i.e., those responsible for the topological entanglement length *N*_e_ (see Figure 6)] are completely equivalent for the two architectures. Together with the finding (Figure 5) that knots play a marginal role in determining *N*_e_, this result reinforces the important result of this work: that the physics of the polymer entanglement length *N*_e_ can be captured by only two-chain links.

Finally, while this work is mostly focused on understanding the relation between the rheological entanglement of the melt and the microscopic topological state of its constituent chains, model conformations of randomly knotted and concatenated rings can be adopted^25^ to understand the mechanisms of synthesis of so-called Olympic gels, namely polymer gels made of randomly linked rings like those now realized by using DNA and cutting restriction enzymes.^55^ In particular, the possibility of performing fine-tuning of the fiber parameters allows one to foresee in great detail how one can benefit from the topological properties of the gel and design materials with certain specificities. For instance, a byproduct of this work concerns how the polymer length, combined with the bending stiffness of the chain, influences the topology of the resulting structure. Depending on κ_bend_, there is a different critical *N* for which a percolating network of concatenated rings appears (Figure S6); in particular, longer and stiffer rings typically produce more robust networks. Moreover, depending on *N* and κ_bend_, the networks are constituted by a complex zoo of catenation motifs: Hopf links, which are the most abundant for all considered values of *N* and κ_bend_ [Figure 3 (left panel) and Figure S5], some more complex links for which GLN = 0 (e.g., Whitehead link) and |GLN| > 1, or links involving three-chain structures whose abundances increase with *N* and κ_bend_ [see Figure 3 (right panel) and Figure 4]. These considerations highlight the topological complexity that may arise in Olympic gels consisting of strand-crossing rings as in ref (55) and how topology can be finely regulated by controllable external parameters such as *N* and κ_bend_.

## Supporting Information Available

Mean-square gyration radii for unknotted and nonconcatenated rings versus randomly knotted and concatenated rings, bond-vector correlation functions, average minimal contour length for rings of a fixed knot type, fractional population of each knot type, mean number of two-chain links per ring as a function of the absolute Gauss linking number and for different ring contour lengths, probability of a ring being nonconcatenated and mean fraction of rings belonging to the largest connected component, and unknot probability and mean number of two-chain links in melts of linear chains (PDF)
