# Supplementary material for: Topological Analysis and Recovery of Entanglements in Polymer Melts
Source: Macromolecules. 2023 Apr 18;56(9):3354–62. doi: 10.1021/acs.macromol.3c00278 (PMC10173697; doi:10.1021/acs.macromol.3c00278)
Supplement: Supplementary file 1 — ma3c00278_si_001.pdf [file ma3c00278_si_001.pdf]

# – Supporting Information –
Topological analysis and the recovery of entanglements in polymer melts

Mattia Alberto Ubertini and Angelo Rosa*
Scuola Internazionale Superiore di Studi Avanzati (SISSA), Via Bonomea 265, 34136 Trieste, Italy

Table of Content

Fig. S1 .... page S2
Fig. S2 .... page S3
Fig. S3 .... page S4
Fig. S4 .... page S5
Fig. S5 .... page S6
Fig. S6 .... page S7
Fig. S7 .... page S8
References .... page S9

* anrosa@sissa.it

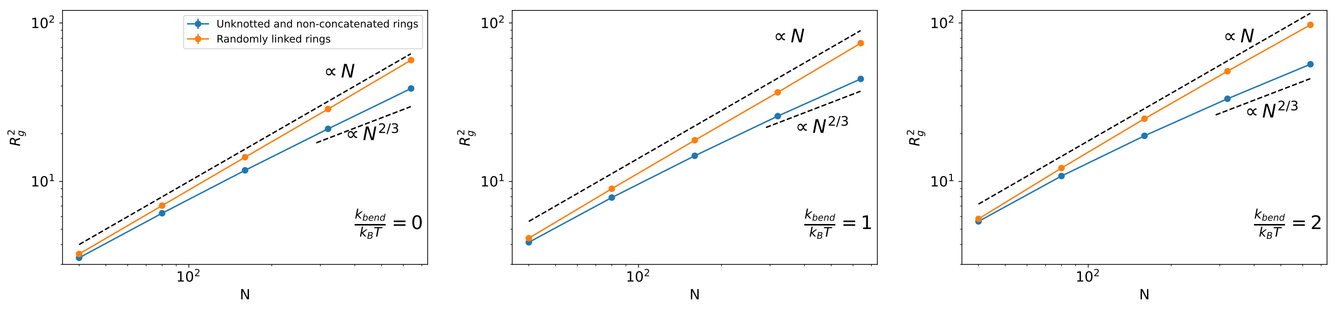

FIG. S1. Mean-square gyration radius $\langle R_g^2 \rangle$ for melts of unknotted and non-concatenated rings (data from Ref. [1]) and randomly linked rings as a function of the total number of monomers per ring, N. Panels from left to right are for bending stiffnesses $\kappa_{\rm bend}/(k_B T) = 0, 1, 2$ (see label).

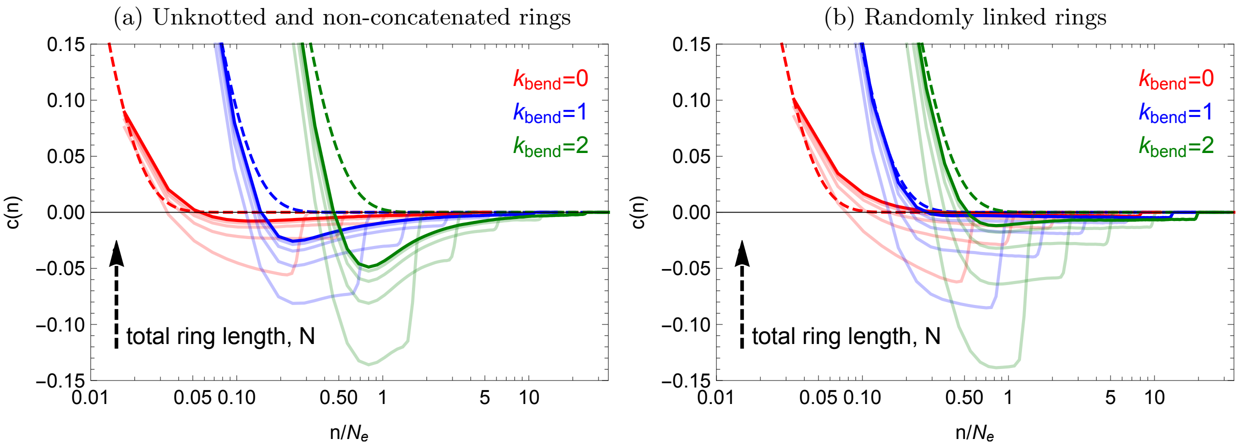

FIG. S2. Bond-vector correlation function $c(n)$ (Eq. (2) in the main paper) as a function of the effective monomer length, n/N_e, normalized with respect to the entanglement length N_e. The l.h.s. and r.h.s panels are for (a) melts of unknotted and non-concatenated rings (data from our previous work Ref. [1]) and (b) randomly linked rings studied in this paper. Lines of equal color are for the same chain stiffness (κ_{bend} in units of $k_B T$, see legend), full colors are for the longest rings ($N = 640$), while lines in fainter colors are for chains of shorter contour lengths (see arrow's direction). The long-dashed lines correspond to the exponential decay typical of linear polymers with local stiffness, *i.e.* $c(n) = \langle \cos\theta \rangle^n$. The values for $\langle \cos\theta \rangle$ and N_e used in panels (a) and (b) are from, respectively, Ref. [1] and the present work (see Table I and Sec. 3.4 in the main text).

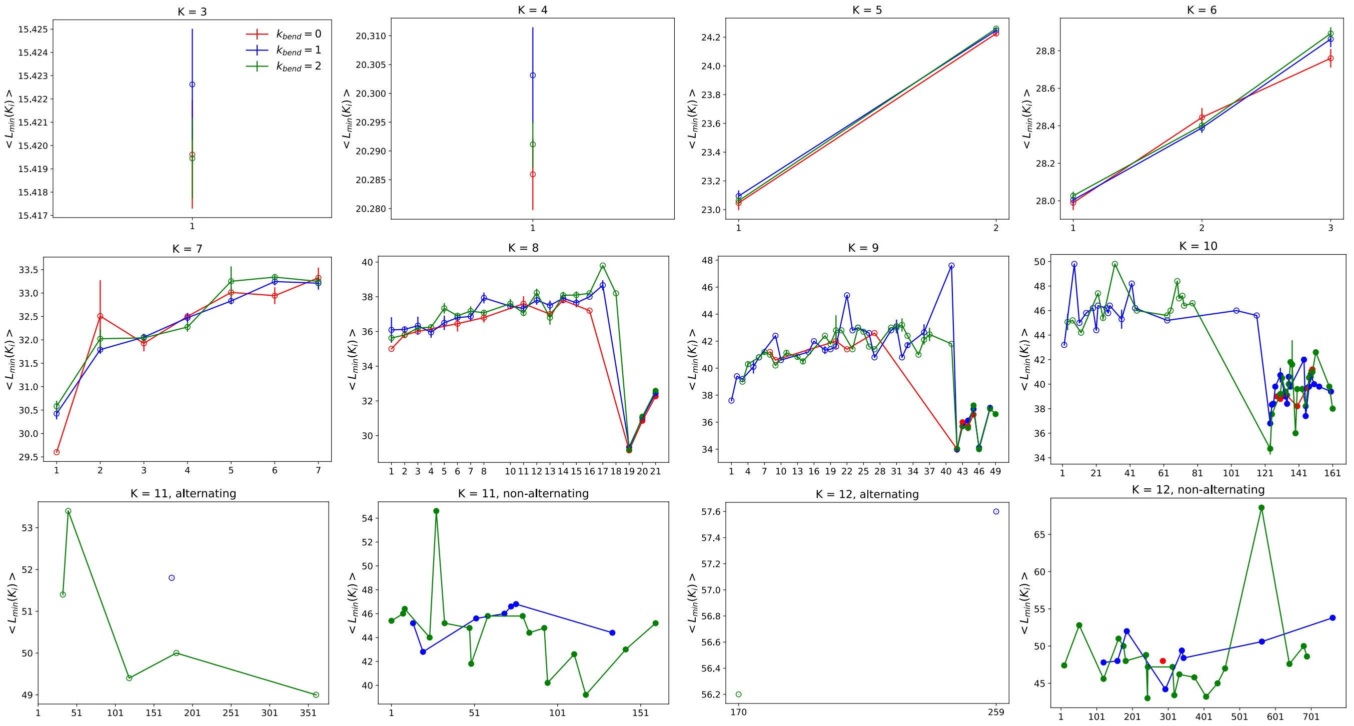

FIG. S3. Average ring minimal contour length (with error bars), $\langle L_{\min}(K_i)\rangle$, computed for each knot type. The labels on the x-axis are for each possible knot at the given K; for $K \geq 9$, the labels (starting from 1) appear with regular spacing for reasons of space (except the panel "$K = 12$, alternating" where only two knots have been detected). As in Figs. 3 and 4 in the main text, empty/full circles are for alternating/non-alternating knots. As in the rest of the paper (see Sec. 2.3.1 in the main text). knots with $K \leq 10$ crossings are named according to the Rolfsen's convention while knots with $K = 11$ and $K = 12$ crossings are conventionally [2] split into alternating, $K_{\rm a\_i}$, and non-alternating, $K_{\rm n\_i}$, ones with the ordered index $i \geq 1$ in both cases. Data with no error bars are for rare knot types, which occur only once in the generated melt conformations. The results shown here are for rings with $N = 640$ monomers and different values of the bending stiffness, $\kappa_{\rm bend}$.

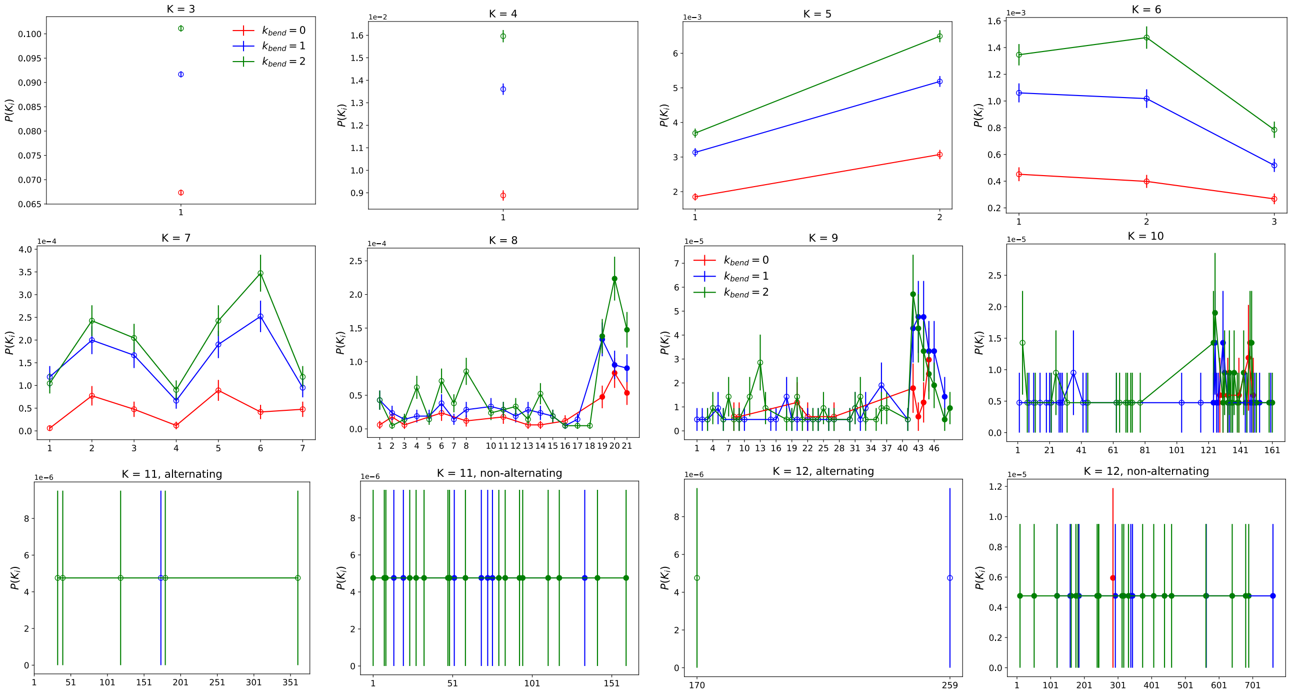

FIG. S4. Fractional population (with error bars), $P(K_i)$, computed for each knot type. Notice that the values on each y-axis have to be multiplied by the power-law reported on the top left corner of the corresponding panel. Symbols, labels and notation are as in Fig. S3. Large error bars are due to the limited size of the relative sample.

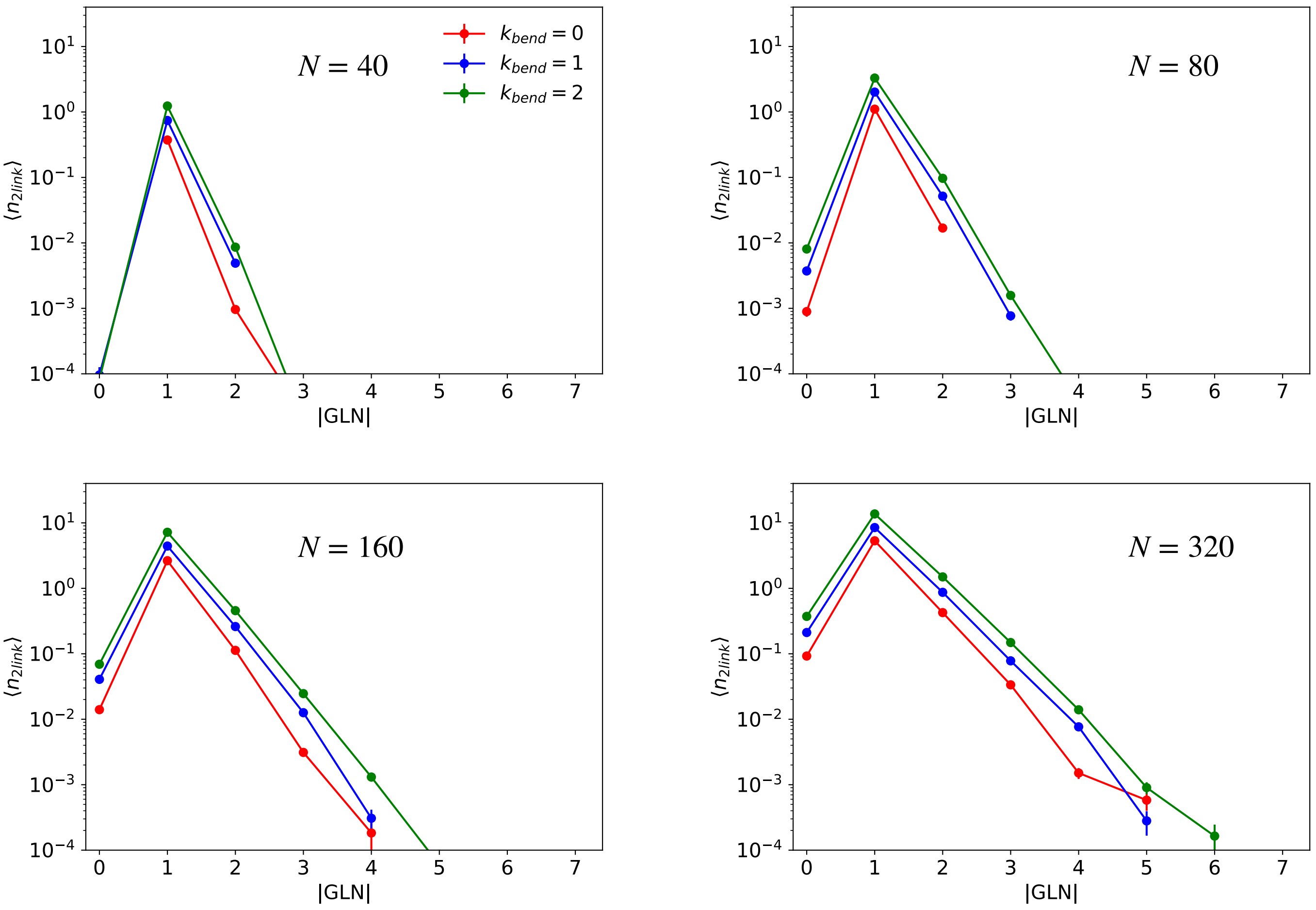

FIG. S5. $\langle n_{\rm 2link}(|{\rm GLN}|)\rangle$, mean number of 2-chain links per ring with absolute Gauss linking number |GLN|. Results for rings with N monomers (to be compared to the results for $N = 640$ reported in the l.h.s. panel of Fig. 3 in the main text).

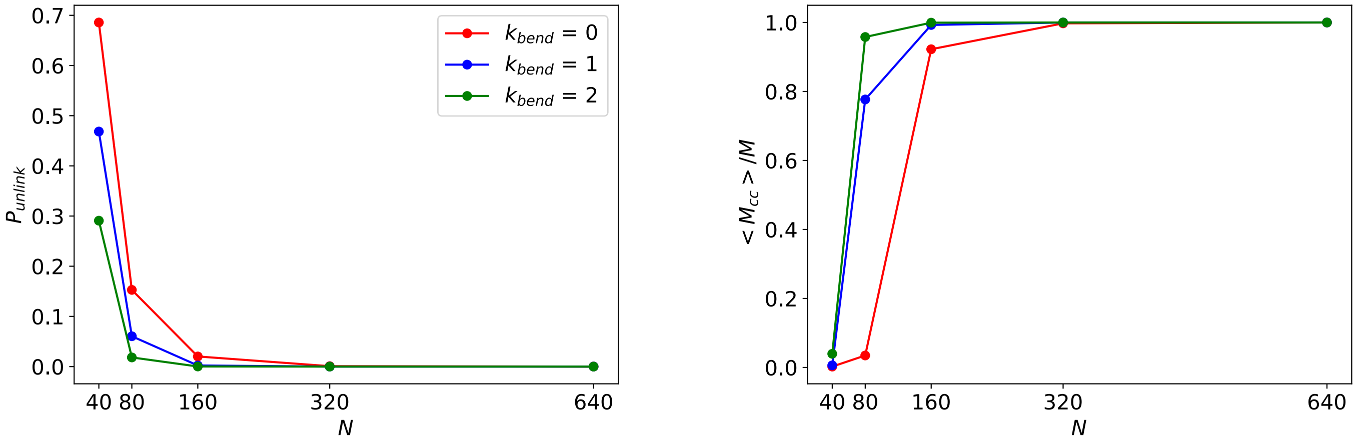

FIG. S6. (Left) Probability, P_{unlink}, that a ring is *not* concatenated to any other ring of the system as a function of N and κ_{bend}. (Right) Mean fraction of rings, $\langle M_{\text{cc}} \rangle / M$, belonging to the *largest connected component* of chains in the melt as a function of N and κ_{bend}.

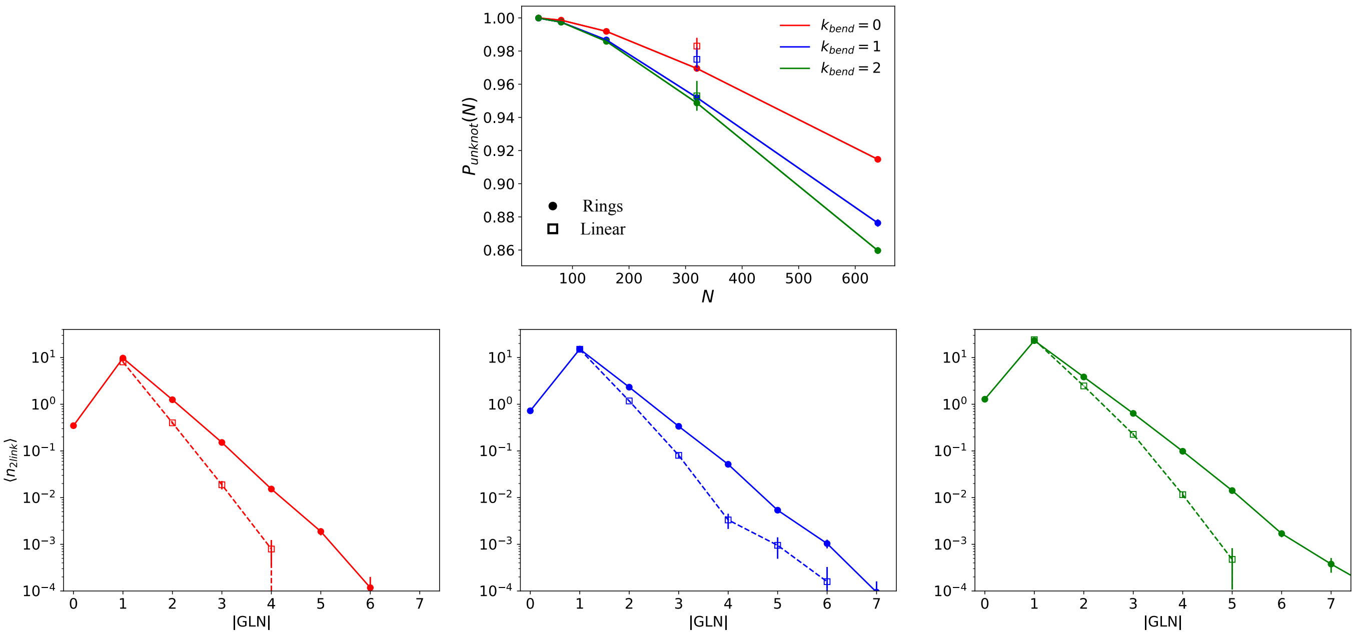

FIG. S7. (Top row) Comparing the unknot probability, P_{unknot}, for *ring* melts (circles / solid lines, same as in Fig. 2 (left panel) in the main text) *vs.* the one for *linear* melts with $N = 320$ monomers per chain (open squares, details on the simulation runs can be found in [1]). The abundance of knots in linear melts have been quantified by using *Topoly* [2], with the following prescription: (i) each of the two free end monomers of a single linear chain is connected to a randomly chosen point on a large spherical surface containing the whole chain; (ii) those added points are joined together through an arc on the sphere; (iii) the Jones polynomial associated to the now closed curve is calculated. This (stochastic) procedure is repeated several times, then the knot associated to the chain is the one corresponding to the *most probable* Jones polynomial between those calculated for the distinct random closures. (Bottom row) Comparing the mean number of 2-chain links per polymer with absolute Gauss linking number |GLN|, $\langle n_{\text{2link}}(|\text{GLN}|)\rangle$, for the same linear melts (open squares / dashed lines) and for ring melts with $N = 640$ monomers per chain (circles / solid lines, same as in Fig. 3 (left panel) in the main text). Notice that the measured GLN (see Eq. (3) in the main text) between two linear chains is, typically, a *real* – instead of an *integer* – number: then, to ensure a fair comparison to the rings' result, we round the GLN of linears to the corresponding *nearest integer*. Notice that since we are only computing GLN and not the Jones polynomial (see Sec. 3.2 in the main text), we can not detect links with GLN = 0.

[1] M. A. Ubertini, J. Smrek, and A. Rosa, Macromolecules **55**, 10723 (2022).
[2] P. Dabrowski-Tumanski, P. Rubach, W. Niemyska, B. A. Gren, and J. I. Sulkowska, Briefings in Bioinformatics **22**, bbaa196 (2021).
